# Event-Related Potentials Reveal the Impact of Conflict Strength in a Numerical Stroop Paradigm

**DOI:** 10.3390/brainsci13040586

**Published:** 2023-03-30

**Authors:** Nydia Vurdah, Julie Vidal, Arnaud Viarouge

**Affiliations:** Institute of Psychology, Université Paris Cité, LaPsyDÉ, CNRS, F-75005 Paris, France

**Keywords:** attention to number, numerical Stroop, ERPs, congruency effects

## Abstract

Numerical cognition provides an opportunity to study the underlying processes of selective attention to numerical information in the face of conflicting, non-numerical, information of different magnitudes. For instance, in the numerical Stroop paradigm, participants are asked to judge pairs of Arabic digits whose physical size can either be congruent (e.g., 3 vs. 5) or incongruent (e.g., 3 vs. 5) with numerical value. Congruency effects when deciding which of the two digits is numerically larger are thought to reflect the inhibition of the irrelevant physical size. However, few studies have investigated the impact of the salience of the irrelevant non-numerical information on these congruency effects and their neural substrates. EEG was recorded in 32 adults during a numerical Stroop task with two levels of salience (low, high) of the irrelevant size dimension. At the behavioral level, we observed larger congruency effects in the high salience condition (i.e., when the difference in size between the two digits is larger). At the neural level, at centro-parietal electrodes, we replicated previous studies showing a main effect of congruency on event-related potential (ERP) amplitudes between 280 and 370 ms post-stimulus, as well as a main effect of salience around 200 ms post-stimulus. Crucially, congruency and salience interacted both between 230 and 250 ms (P2), and between 290 and 340 ms (P3). These results provide support for separate processes underlying the increase in congruency effect, which can be attributed to higher demands in both the inhibition of the irrelevant dimension, and the attention to the relevant numerical information.

## 1. Introduction

The number of objects in a set is invariant in spite of changes in perceptive properties of the set, such as how spread apart or how large the objects are. Since the seminal work of Jean Piaget on number conservation [1], research in numerical cognition has shed light on the cognitive processes underlying the understanding of number as an invariant. In particular, a body of research has emerged investigating what has been referred to as “attention to number” (AtN, [2]). AtN can be defined as the ability to process numerical information in the face of non-numerical, possibly conflicting, information of magnitudes.

Indeed, many studies have reported congruency effects when comparing two sets of objects on the basis of their quantities [3]. In general, performance is lower when the numerical and non-numerical information are incongruent (e.g., when the set containing more objects presents smaller objects), than when they are congruent (e.g., when the set containing more objects presents larger objects). These congruency effects between numerical and non-numerical information can also be observed in the symbolic domain. For instance, in the numerical Stroop paradigm, participants are asked to judge pairs of Arabic digits whose physical size can either be congruent (e.g., 3 vs. 5) or incongruent (e.g., 3 vs. 5) with their numerical value [4].

Most studies have interpreted the number/size congruency effects as reflecting the role of inhibitory control processes in blocking the irrelevant, non-numerical information when performing numerical comparison. Using priming paradigms, recent studies have indeed provided evidence for such a role, both in the non-symbolic [5,6] and in the symbolic domains [7,8]. However, and as suggested by Wilkey and colleagues [2], AtN likely encompasses both inhibitory control processes to block the conflicting information, and attentional processes to reorient attention towards the relevant numerical information. Regarding the latter, some studies have recently examined the impact of the salience of the irrelevant non-numerical information during numerical comparison tasks. For instance, early school-aged children’s performance when judging whether two rows of tokens contain the same or different numbers of objects evolves gradually as a function of the difference in length between the two rows [9]. The greater the difference in salience, the more difficulty children have in making judgments based on number. This result can be interpreted both as an increase in the inhibitory control demand of the task when the difference in length is more salient, or as an increase in difficulty in orienting attention towards the relevant numerical dimension. In the context of the symbolic numerical Stroop task, manipulating the salience of the irrelevant physical size dimension has been shown to impact the congruency effect, providing evidence for the role of selective attention to the relevant dimension [10].

FMRI studies have identified overlapping brain regions in the parietal lobe when processing both numerical and non-numerical dimensions of magnitude [11,12]. Regarding EEG, several studies have investigated the ERPs indexing the number/size congruency effects. In a symbolic numerical Stroop paradigm, Szűcs and Soltész [13,14] observed that interference effects between number and size yielded an early ERP component appearing over occipito-parietal sites within a 150–250 ms time-window after stimulus onset and a late ERP component within the 340–430 ms time frame, with a negative peak over centro-parietal regions and a positive peak over frontal regions. In their study, using both non-symbolic and symbolic numerical Stroop tasks, Gebuis and colleagues [15] observed congruency effects on a P3 component peaking within a 300–500 ms time window after stimulus onset in parietal regions, with a less positive, later P3 in incongruent compared to congruent trials. While these studies allowed better characterization of the neural substrates of number/size congruency effects, they have not yet systematically investigated the impact of conflict strength by manipulating the salience of the irrelevant dimensions of magnitude. In the context of symbolic and non-symbolic numerical comparison tasks, a posterior P2 ERP component (P2p) is thought to index numerical distance (with greater amplitude of the P2p component for smaller numerical distances) [16]. However, other studies have reported an influence of the visual properties of the stimuli on P2p amplitudes during numerical comparison [17]. In research in the field of vision, the P2 ERP component is thought to index selective attention processes [18]. In particular, several studies have reported a decreased P2 amplitude with increasing visual saliency in the context of visual search paradigms [19,20].

Based on previous behavioral studies mentioned above, increasing the salience of the irrelevant dimensions should result in larger congruency effects, due to a larger demand in AtN. However, the question remains open as to whether these larger congruency effects reflect greater demands in inhibitory control, in attentional processes to focus on the relevant dimension, or both. EEG can help us shed light on this question, by analyzing the impact of congruency and salience of the irrelevant dimension of magnitude on the elicited ERP components. First, analyses of the congruency and salience effects over parietal sites will allow us to identify their underlying ERP components. Then, we will investigate the ERPs associated with the modulation of the congruency effect by the salience of the irrelevant dimension. If the increase in conflict strength results in an increase in both inhibitory control and attentional demands, then we should see its impact on ERP waves associated with both the congruency and the salience effects. The EEG studies reviewed above point towards the P3 and P2 components as potential candidates for indexing these effects, respectively.

## 2. Materials and Methods

### 2.1. Participants

We recruited 32 healthy adult participants (26 female, 6 male) aged between 18.39 and 25.80 years old (mean age = 20.54 years, SD = 1.81) from the Psychology undergraduate student population of Paris Cité University (*n* = 27) and through word-of-mouth (*n* = 5). The participants were recruited at random and were unaware of the hypotheses of this study. The skewed sex distribution of our sample reflects the sex distribution of the Psychology undergraduate student population at our university. All the participants were right-handed, fluent in French, had normal or corrected-to-normal vision and had no history of neurological disorders or traumatic injury. This study was approved by the ethical committee of the Paris Cité University (IRB #00012020-83), in line with the Declaration of Helsinki and the Jardé Law in France regarding research involving human participants (RIPH). Participants signed a written informed consent prior to the beginning of the experiment and were given a short explanation of the aims of the study once all the data were collected.

### 2.2. Task and Procedure

**Figure 1:**
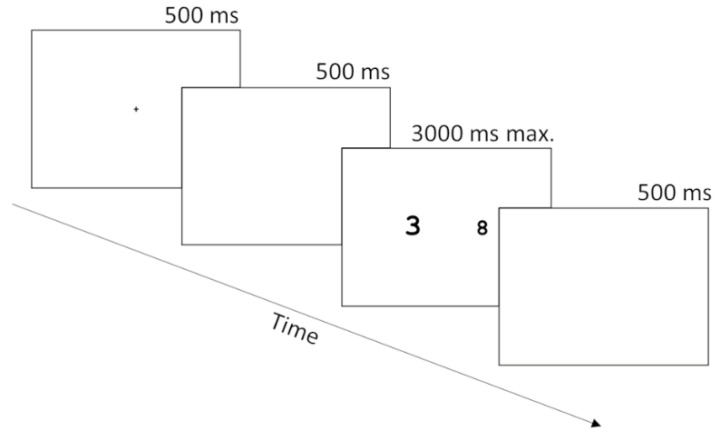
Schematic of a trial in the symbolic numerical Stroop task. The figure shows an example of a number/size incongruent trial with high salience of the irrelevant size dimension.

The participants performed a numerical Stroop task while their behavioral and electrophysiological responses were recorded. The task design was adapted from that of Szűcs and Soltész [14]. The participants were comfortably seated at a distance of approximately 60 cm from a 17-inch LCD monitor linked to a PC computer equipped with E-prime 2.0 professional software (Psychology Software Tools, PST, Pittsburgh, PA, USA. Pairs of Arabic digits were displayed on the left and right sides of the screen and the participants were instructed to respond on the side of the numerically larger digit, by pressing either on the far-left or the far-right button on a 4-button response box. Stimuli consisted of four pairs of Arabic digits ranging from 2 to 8. Two numerical distances were used: distance 1 (2–3 and 7–8) and distance 5 (2–7 and 3–8), in order to vary the material and prevent the participants from learning an automatic strategy. The digits were displayed in black over a white background, centered vertically, and at a horizontal visual angle of 2.6 from the center of the screen. They were presented in bold Courier New font in three different sizes: 42, 48, and 56, which corresponded to occupied vertical visual angles of 2, 2.3, and 2.7, respectively. We used two different physical size ratios between the two digits of the pairs: 0.875 (for font sizes 42 and 48) and 0.75 (for font sizes 42 and 56). Digit pairs with the larger size ratio (smaller difference in font sizes) will be henceforth referred to as the “Low salience” pairs, while pairs with the smaller size ratio (larger difference in font sizes) will be referred to as the “High salience” pairs. Trials were either congruent (the numerically larger value is also the physically larger one) or incongruent (the numerically larger value is the physically smaller one). Neutral trials, whereby both digits were presented in font 42, were also presented, ensuring regular recall of the instruction (comparison on the basis of numerical value) throughout the task. Each trial began with the presentation of a central fixation cross for 500 ms, followed by a white screen for another 500 ms. The pair of digits then appeared until the participant’s response, for a maximum of 3 s. Following the participant’s response, or after 3 s of stimulus presentation, a 500 ms white screen was presented, after which the following trial began (Figure 1).

The numerical Stroop task consisted of 24 practice trials followed by 8 experimental blocks of 72 trials each, yielding 576 experimental trials in total. The side of the correct response, the numerical distance, the salience of physical size, and the congruency were counterbalanced across the practice trials, and across the experimental trials of the task. The EEG session lasted approximately 1 h in total for the preparation and installation of the participants, including 20 min for the duration of the numerical Stroop task itself. Participants were invited to take a short break between each block.

### 2.3. Electrophysiological Recordings

Electroencephalograms (EEG) were recorded from a 256-channel HydroCel Geodesic Sensor Net (Electrical Geodesics Inc., Eugene, OR, USA) containing electrodes imbedded in small sponges soaked in a potassium chloride saline solution. Continuous EEGs were acquired through a DC amplifier (Net Amps 300 1.0.1, EGI) and digitized at a sampling rate of 500 Hz. A common reference at the vertex was used during acquisition, and electrode impedances were kept below 100 kΩ. Eye-blinks and eye-movements were monitored via pairs of channels included in the net. The processing steps described below were performed using EEGLAB [21]. Only the EEG data for the correctly responded trials were analysed.

The raw EEG data were passed through a high-pass filter of 0.5 Hz and a low-pass filter of 30 Hz.

**Figure 2:**
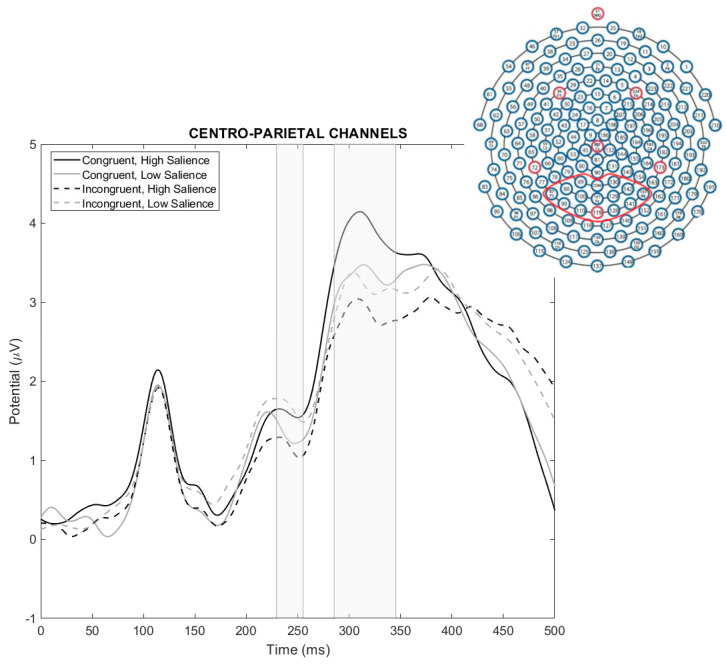
Grand averaged ERP waveforms for each of the four congruency × salience experimental conditions, over centro-parietal channels (circled in red in the electrodes schematic, top right corner). Grayed areas correspond to time periods showing a significant congruency × salience interaction.

Channels covering the lower sides of the face (temples) and lower back of the head were removed from the channel array as they often had high impedances and were not pertinent for our study. Thus, further preprocessing was performed on the 174 channels evenly distributed on the scalp (Figure 2). Bad channels and artifacts were removed from continuous EEG data using Artifact Subspace Reconstruction (ASR, implemented in an EEGLAB plugin, i.e., clean_rawdata [22]). Spherical interpolation was used to replace bad channels, and the signal from all electrodes was re-referenced to the average. The continuous EEG data were then segmented from −200 ms to +1000 ms relative to the onset of stimulus. The epochs were baseline corrected using the mean pre-stimulus voltage in the 200 ms pre-stimulus period. For each participant, numerical distances 1 and 5 were pooled together, and segments were binned into the four different experimental conditions: (congruent, incongruent) × (low salience, high salience).

Six out of the 32 participants (5 female, 1 male) were excluded from the analyses because of too many artifacts in their EEG recording, resulting in less than 200 epochs across all experimental conditions. Statistical analyses were then performed on the remaining 26 participants (21 female, 5 male), whose EEG recordings showed at least 250 epochs across the conditions.

## 3. Results

### 3.1. Behavioral Analyses

As expected in this task, the participants’ accuracy rates were high (mean = 0.96, SD = 0.19), and behavioral analyses were conducted on the reaction times. We ran a repeated-measures ANOVA analysis on reaction times for correctly responded trials, with salience (low, high) and congruency (congruent, incongruent) as independent variables. We observed a main effect of congruency, *F*(1, 31) = 125.28, *p* < 0.001, η^2^_p_ = 0.8, with higher reaction times for incongruent trials (mean = 498.1 ms, SD = 160.27) than for congruent trials (mean = 442.04 ms, SD = 148.33). There was also a main effect of salience, *F*(1, 31) = 5.87, *p* = 0.02, η^2^_p_ = 0.16, with higher reaction times when the salience of physical size was high (mean = 472.17 ms, SD = 151.46) than for low salience (mean = 466.22 ms, SD = 161.66). Importantly, we found a significant congruency × salience interaction, *F*(1, 31) = 45.39, *p* < 0.001, η^2^_p_ = 0.59, with a larger average amplitude of the congruency effect for high salience trials (72.86 ms) than for low salience trials (34.23 ms), in line with our hypotheses and previous studies [10].

### 3.2. ERP Analyses

Figure 2 displays the ERP waveforms of each congruency (congruent, incongruent) × salience (low, high) trial condition, at selected electrodes over the centro-parietal site (Left Hemisphere: channels 87 (P3), 88, 89, 99, 100, 110; Right Hemisphere: 128, 129, 130, 141, 142, 153 (P4); and central channels 101 (Pz) and 119). Grand average ERP waveforms showed a positive wave occurring from 200 ms, which we thereafter refer to as the P2 component, and a positive wave occurring from 250 ms, which we thereafter refer to as the P3 component.

Two-way ANOVA analysis on ERP amplitudes with salience (low, high) and congruency (congruent, incongruent) as independent variables were performed using EEGLAB, at each time sample and each selected electrode site. This analysis allowed us to highlight the time periods when ERP differences were significant (*p* < 0.05) during several consecutive time samples. We observed a main effect of congruency on ERP amplitudes between 280 and 370 ms post-stimulus, replicating previous EEG studies of the numerical Stroop task [15], as well as a main effect of salience around 200 ms post-stimulus. Crucially, congruency and salience interacted both between 230 and 250 ms (P2), and between 290 and 340 ms (P3, Figure 2).

In order to provide converging evidence, we conducted a second type of analysis, similar to that conducted on the behavioral data. We pre-defined time windows centered around the peak of the P2 and P3 components of interest: 200–250 ms for the P2 component, and 250–350 ms for the P3 component. Then, for each ERP component, we ran a 2 salience (low, high) × 2 congruency (congruent, incongruent) repeated measures ANOVA on the mean amplitudes in the corresponding time windows.

For the P2 component, repeated-measures ANOVA confirmed a marginally significant main effect of salience, *F*(1, 25) = 3.68, *p* = 0.07, η^2^_p_ = 0.13, in the absence of a significant congruency effect, *F*(1, 25) = 0.04, *p* = 0.85, η^2^_p_ = 0.001. Importantly, in this time window, mean amplitudes of the P2 component showed a significant congruency × salience interaction, *F*(1, 25) = 4.6, *p* = 0.04, η^2^_p_ = 0.16. Post-hoc comparisons revealed a significant effect of salience for incongruent trials with a lower mean amplitude in high salience (mean = 1.14 μV) compared to low salience trials (mean = 1.63 μV, *p* = 0.04), and no effect of salience for congruent trials (low salience: mean = 1.38 μV, high salience: mean = 1.44 μV, *p* = 0.71).

For the P3 component, repeated-measures ANOVA showed a significant main effect of congruency, *F*(1, 25) = 9.19, *p* < 0.01, η^2^_p_ = 0.27, no significant effect of salience, *F*(1, 25) = 1.05, *p* = 0.32, η^2^_p_ = 0.04, and a significant congruency × salience interaction, *F*(1, 25) = 8.02, *p* < 0.01, η^2^_p_ = 0.24. Post-hoc comparisons showed a significant effect of congruency for high salience trials, with higher mean amplitude in congruent (mean = 3.29 μV) than in incongruent trials (mean = 2.42 μV, *p* < 0.001), and no effect of congruency for low salience trials (congruent: mean = 2.79, incongruent: mean = 2.73, *p* = 0.76).

## 4. Discussion

When comparing the numerical value of two Arabic digits, participants are faster when the physical size of the digits is congruent with their numerical value than when it is incongruent. As shown in previous studies [10], this congruency effect is larger when the salience of the irrelevant size dimension is larger, i.e., when the conflict between numerical and non-numerical information is stronger. This increase in the amplitude of the congruency effect could reflect a greater demand in inhibitory control to block the irrelevant information of size, but also greater difficulty in orienting one’s attention to the relevant numerical information. The ERP analyses revealed that the P3 amplitude over centro-parietal channels decreased in the incongruent condition. The P3 component is thought to reflect cognitive control, including inhibitory control processes [23], generated at the level of the posterior parietal cortex [24]. This result replicates previous studies on the neural substrates of number/size congruency effects [15]. Interestingly, we observed a distinct ERP component associated with the main effect of salience of the irrelevant information, independently from the number/size congruency. The amplitude of the P2 component was larger for low salience than for high salience trials. This is in line with studies showing an impact of visual features of the stimuli on the amplitude of a posterior P2 component during numerical judgment tasks, beyond the effect of numerical distance [17]. Although less well understood than the P3 component, the P2 component has been studied in particular in the context of visual search paradigms and is thought to be related to processes of selective attention [18]. In line with our results, several studies have reported a decreased P2 amplitude with increasing visual saliency [19,20]. Crucially, both the P2 and the P3 components were associated with the interaction between congruency and salience in our study. The difference in amplitude in the P3 component between congruent and incongruent trials was increased in the high salience condition, possibly reflecting the higher inhibitory control demand. Regarding the P2 component, post-hoc tests showed a significant effect of the salience of the irrelevant information only in the incongruent condition, with a greater amplitude in the low salience condition. In the context of incongruent trials, the lower amplitude of the P2 component could index a greater demand in selective attention to the relevant information. Further studies are needed to better understand the underlying processes of the posterior P2 component in the context of numerical judgment tasks, and in particular to jointly study the impact of the salience of the relevant and irrelevant information of magnitude.

While our sample size corresponds to the standard usually used in similar EEG studies, and exceeds those used in previous studies of the neural correlates of symbolic numerical Stroop ([13,14,15]: *n*s < 18), it could be argued that our results are potentially impacted by an imbalance in the sex of our participants. Specifically, our results could potentially be generalizable only to a population of female adults. As mentioned previously, this skewed distribution was a direct consequence of our method of recruitment. To our knowledge, no study to date has examined sex differences in the specific context of the symbolic numerical Stroop task. Further studies are needed to investigate potential sex differences in the underlying processes of AtN and the mechanisms of their development.

Finally, another limitation of our study is that, although the P3 component has generally been associated with cognitive control, the latter could comprise both inhibitory control and selective attention processes. Nevertheless, our study design allows us to investigate the ERP waves separately associated with the congruency effect on one hand, and the effect of salience on the other hand, revealing distinct related components.

## 5. Conclusions

Altogether, these results help shed light on the underlying processes of Attention to Number (AtN). They provide support for separate processes underlying the increasing congruency effect with increasing conflict strength, which can be attributed to higher demands in both the inhibition of the irrelevant dimension, and the attention to the relevant numerical information. fMRI studies using a similar paradigm systematically manipulating the salience of the irrelevant information could help identify the brain regions involved in these processes. AtN is thought to be a crucial ability in the development of numerical cognition [25]. Indeed, the development of the precision of children’s numerical representation relies, at least in part, on the development of their ability to process numerical information in the face of conflicting visual information. Further studies in younger participants will be needed to investigate the developmental trajectories of these underlying processes and their neural substrates.

## Data Availability

Publicly available datasets were analyzed in this study. This data can be found here: [https://osf.io/pbeku/?view_only=3accd8b57d8f406a9fb534afb3c0a4a7, accessed on 28 February 2023].
